# Apnea test for the diagnosis of brain death in a patient undergoing extracorporeal membrane oxygenation

**DOI:** 10.5935/0103-507X.20200077

**Published:** 2020

**Authors:** Viviane Cordeiro Veiga, Ligia Maria Coscrato Junqueira Silva, Erica Regina Ribeiro Sady, Priscila Valente Fernandes, Salomón Soriano Ordinola Rojas

**Affiliations:** 1 BP - A Beneficência Portuguesa de São Paulo - São Paulo (SP), Brazil.; 2 Brazilian Research in Intensive Care Network (BRICNet) - São Paulo (SP), Brazil.

**Keywords:** Brain death, Apnea, Extracorporeal membrane oxygenation, Morte encefálica, Apneia, Oxigenação por membrana extracorpórea

## Abstract

Extracorporeal membrane oxygenation is used as extracirculatory support for the care of patients with severe and reversible cardiac and/or respiratory failure. Neurological complications may be related to the procedure. Given the unfavorable neurological evolution and the need to perform a brain death protocol, the performance of an apnea test in this context remains a challenge. We report the use of an apnea test for the diagnosis of brain death post-cardiac surgery in a patient receiving venoarterial extracorporeal membrane oxygenation.

## Abstract (pt)

A oxigenação por membrana extracorpórea é utilizada como suporte extracirculatório para a assistência de pacientes em severa e reversível falência cardíaca e/ou respiratória. Complicações neurológicas podem estar relacionadas ao procedimento. Diante da evolução neurológica desfavorável e da necessidade de realização de protocolo de morte encefálica, permanece um desafio a realização de teste de apneia nesse contexto. Relatamos o caso de teste de apneia para diagnóstico de morte encefálica em pós-operatório de cirurgia cardíaca em paciente utilizando oxigenação por membrana extracorpórea venoarterial.

## INTRODUCTION

Extracorporeal membrane oxygenation (ECMO) is increasingly used to provide extracirculatory support for patients with severe and reversible cardiac and/or respiratory failure.^(1)^ Among the related complications, those of neurological etiology are not uncommon, although their impact has been recognized only in recent years.^(2)^ Related neurological complications include cognitive deficits, seizures, embolizations, bleeding, and cerebral hypoxia related to low oxygen flow, with reported rates ranging from 13% to 67%.^(2,3)^

Given the records of brain death (BD) in patients undergoing ECMO, publications describing the diagnostic evaluation process, specifically, the execution of the apnea test, in this particular group are not only variable but scarce since the exchange of gasses is totally or partially controlled by the extracorporeal circulation device and is maintained despite the absence of ventilatory autonomy.^(1)^ Therefore, the diagnosis of BD in this group of patients is a challenge and requires knowledge about the physiological interaction between the organic (patient) and mechanical (ECMO) systems and expertise in the management of device parameters.

We report the use of an apnea test for the diagnosis of BD in a patient undergoing ECMO.

## CASE REPORT

A 31-year-old woman was admitted to the intensive care unit (ICU) in the immediate postoperative period after mitral and aortic valve replacement (biological prosthesis), tricuspid valvuloplasty, enlargement of the aortic ring, and amputation of the left auricle. The surgical procedure had a 7-hour duration, 225 minutes of which were performed under extracorporeal circulation. The patient had a history of rheumatic fever, lupus, previous mitral valve replacement (2010) and aortic valve replacement (2016) and severe pulmonary hypertension.

During the intraoperative period, the patient developed pulmonary hypertension, acute *cor pulmonale* and cardiogenic shock, which required high doses of vasoactive drugs in the ICU, including endovascular and inhaled pulmonary vasodilators (nitric oxide), and the application of protective parameters during invasive mechanical ventilation (IMV) (Table 1). The patient was had a severe overall condition and developed multifactorial renal failure (systemic inflammatory response syndrome, increased extracorporeal circulation time and decreased arterial blood flow: “prerenal pattern”), and renal replacement therapy in the form of continuous venovenous hemofiltration was indicated.

**Table 1:** Evolution of clinical, ventilatory, hemodynamic, and laboratory parameters

	Pre-ECMO	Day 1	Day 2	Day 3	Day 4	Day 5	Day 6	Day 7
Invasive mechanical ventilation								
Ventilatory mode	A/C-CPV	A/C-CPV	A/C-CPV	A/C-CPV	A/C-CPV	A/C-CPV	A/C-CPV	A/C-CPV
Peak pressure (cmH_2_O)	28	30	41	41	39	45	41	41
PEEP (cmH_2_O)	8	5	5	5	8	10	10	8
FiO_2_	0.4	0.5	0.7	0.7	0.85	0.85	1	0.85
Tidal volume (mL)/predicted weight (kg)	6	3	3	3	3	3	3	4
Respiratory rate	20	24	24	24	20	20	20	22
ECMO-VA								
Flow (L/minute)		2.21	3.00	3.00	3.00	2.30	2.29	2.22
RPM		2.02	2.60	2.62	2.64	2.24	2.22	2.22
Sweeper flow (L/minute)		3.0	3.5	3.5	3.5	3.5	3.5	3.5
FiO_2_		0.4	0.7	0.7	0.7	0.7	0.9	0.7
Temperature (ºC)		35.4	35.4	35.6	35.5	35.5	35.5	35.6
ACT		203	171	157	145	170	201	200
Arterial blood gas analysis								
pH	6.98	7.34	7.47	7.39	7.47	7.43	7.31	7.45
PaO_2_ (mmHg)	74	297	99	144	209	180	151	191
PaCO_2_ (mmHg)	85	23	43	40	43	39	50	37
SaO_2_	87	96	97	98	99	99	95	96
EB (mEq/L)	-12.3	-7.7	7.1	-0.2	6.3	1.8	-1.1	1.8
PaO_2_ (mmHg)/FiO_2_	185	199	243	205.7	298.6	257	167.7	272.8
Laboratory								
Lactate (mg/dL)				17	21	19	15	17
Hemoglobin (g/dL)				8.4	7.8	8.8	7.1	7.1
Platelets (/mm^3^)				52.000	38.000	40.000	28.000	33.000
Scores								
Lung injury score	2.75	2.75	2.75	2.5	2	2.25	3.25	2.5

ECMO - extracorporeal membrane oxygenation; A/C-CPV - assisted/controlled constant pressure ventilation; PEEP - positive end-expiratory pressure; FiO_2_ - inspired oxygen fraction; ECMO-VA - venoarterial extracorporeal membrane oxygenation; RPM - rotations per minute; ACT - activated coagulation time; PaO_2_ - arterial oxygen pressure; PaCO_2_ - partial pressure of carbon dioxide; SaO_2_ - arterial oxygen saturation; EB - excess base.

**Figure 1:**
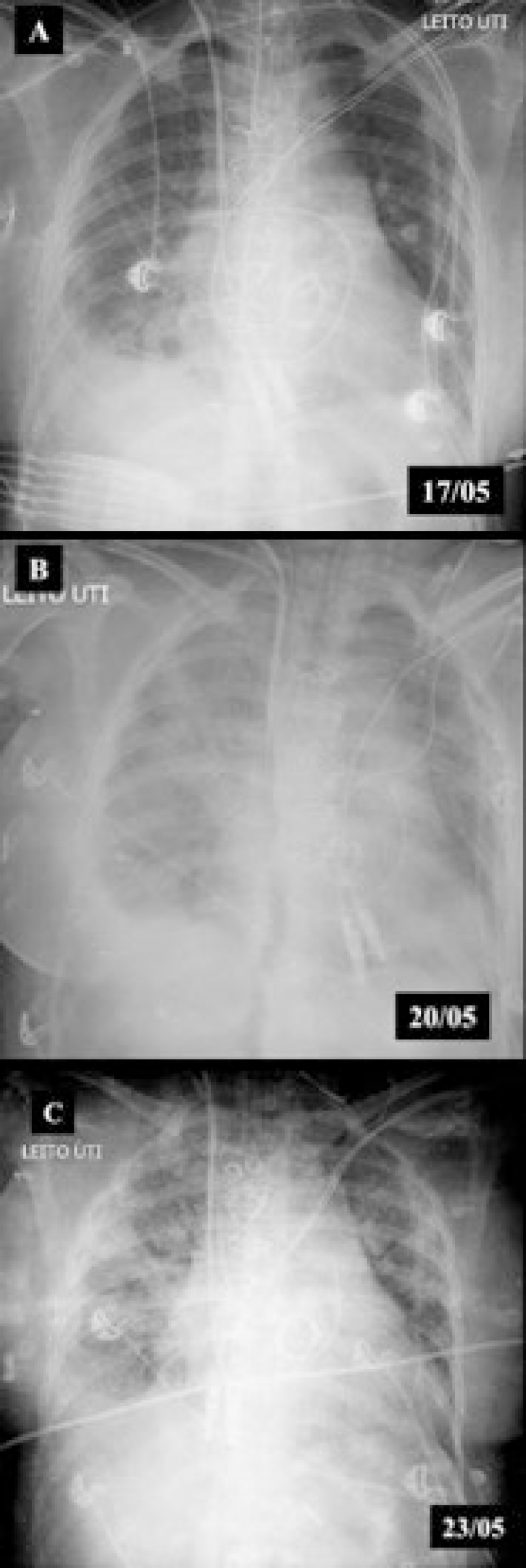
Evolution of chest radiography during hospitalization. (A) Immediate postoperative period (pre-extracorporeal membrane oxygenation); (B) Day 1 - venoarterial extracorporeal membrane oxygenation; (C) Day 4 - venoarterial extracorporeal membrane oxygenation.

After 60 hours without clinical improvement, especially of the hemodynamic condition, and with progressive deterioration of renal and respiratory function (Figure 1), mechanical circulatory support via venoarterial ECMO (VA-ECMO) was indicated (Table 1). With peripheral femorofemoral cannulation via the left lower limb (LLL), the patient presented acute ischemia that required the drainage cannula to be removed and reinserted into the femoral tract of the contralateral lower limb. In addition, once Harlequin syndrome was identified, it was necessary to perform central cannulation. The patient was sedated with a Richmond agitation and sedation scale (RASS) -5, using propofol and remifentanil for analgosedation. Despite the partial recovery of ventricular function resulting from the management of pulmonary hypertension in the echocardiographic evaluation, 96 hours after the initiation of ECMO, the patient presented mydriatic pupils under analgosedation and concomitant and abrupt worsening of the lung image on chest X-ray, with heterogeneous and bilateral opacification as well as oxygenation parameters (Table 1) that suggested neurogenic pulmonary edema. This, added to the pro-inflammatory stimulus of the synthetic surface of the ECMO circuit, explained the decline.

In such cases, it is recommended that management be performed as for acute respiratory distress syndrome (ARDS) through protective ventilation. However, this was not possible without ECMO support due to the risk of hypercapnia; although hypercapnia is “permitted” in the context of ARDS, it did not seem acceptable in this case of acute neurological dysfunction. For these reasons, ECMO support was maintained despite the improvement of the hemodynamic profile, and the process of weaning from the device that had been started was interrupted.

The drugs were discontinued, and the neurological changes persisted. Cranioencephalic computed tomography showed diffuse cerebral edema (hyperemia due to loss of vascular autoregulation), apparent right parietal laminar subdural hematoma, multiple intraparenchymal hematomas of varying dimensions in the cerebral hemispheres, reduction of the amplitude of the supratentorial system, and effacement of the sulci between the cortical gyri of the cerebral hemispheres.

**Figure 2:**
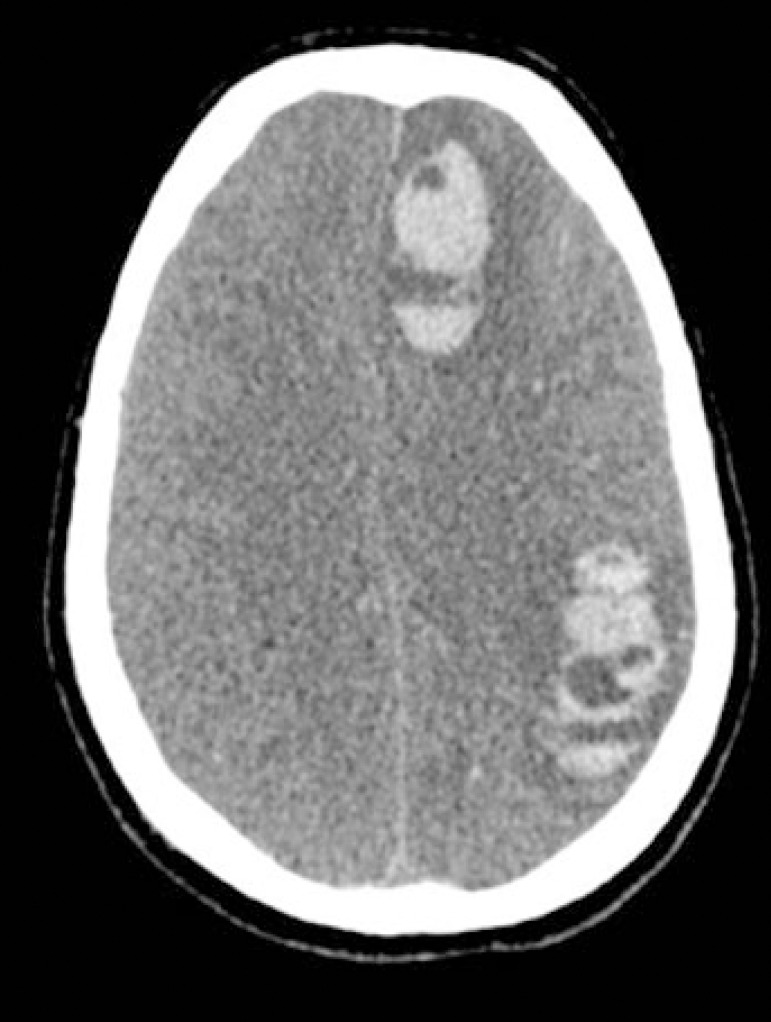
Tomography of the skull obtained on the fifth postoperative day (and fourth post-extracorporeal membrane oxygenation). Signs of diffuse cerebral edema, subdural hematoma, intraparenchymal hematomas, and effacement of the sulci between the cortical gyri of the cerebral hemispheres.

After 24 hours, the findings of neurological dysfunction, including mydriatic and unreactive pupils and loss of other brainstem reflexes, were maintained, except for respiratory drive. A new computed tomography scan (Figure 2) showed the progression of neurological lesions with signs of transtentorial herniation.

After 12 hours of this event, loss of respiratory drive was detected; thus, the procedures for the diagnosis of BD were initiated according to the recommendations of the Resolution of the Federal Council of Medicine (*Conselho Federal de Medicina* - CFM) 2173/2017. The case fulfilled all pre-requirements: identification of the coma-causing lesion through neuroimaging and exclusion of potential factors that could confuse the clinical picture, especially the effects of central nervous system depressant drugs (in this case, remifentanil and propofol); these medications were suspended 36 hours before the application of the apnea test, a duration that the teams considered adequate considering that ECMO can alter the serum concentration of these drugs due to increased volume of distribution in the extracorporeal circuit.^(4)^

In addition, the esophageal body temperature was higher than 35°C (36.5°C), arterial oxygen saturation was above 94%, and systolic blood pressure was greater than or equal to 100mmHg. At that point, two clinical examinations - an apnea test and a complementary test - were performed by trained physicians to determine BD.

In addition to the patient’s state of unreactive and unperceptive coma (Glasgow Coma Scale score of 3), the electroencephalographic examination showed an absence of electrical activity. Along with the other recommended tests, the apnea test was performed under VA-ECMO, fulfilling the necessary prerequisites; the patient presented adequate blood pressure, central temperature and pulse oximetry and the configuration of the VA-ECMO and mechanical ventilator was appropriate, including preoxygenation for 10 minutes with a fraction of inspired oxygen (FiO_2_) of 1.0 in both support devices and the sweep flow reduced to 0.5L/minute (Table 2). The IMV was discontinued, and supplemental oxygen therapy (6L/minute) was instituted via an orotracheal tube. In the first minute, significant hypoxemia was detected despite supplemental oxygen; as recommended, the flow of the VA-ECMO was increased, and physiological parameters recovered to a level acceptable for the test. Strict monitoring for the presence of respiratory movements was performed for 10 minutes, and blood samples for arterial blood gas analysis (pre- and posttest) were collected, which showed an increase in the partial pressure of carbon dioxide (PaCO_2_).

**Table 2:** Parameters of venoarterial extracorporeal membrane oxygenation and blood gas analysis, pre- and post-apnea test

	Apnea test	
	Pre	Post
ECMO-VA		
Flow (L/minute)	2.22	4
RPM	2.22	3.7
Sweeper flow (L/minute)	3.5	0.5
FiO_2_	0.7	1
Temperature (ºC)	36.5	36.5
Hemodynamics		
MAP (mmHg)	82	69
HR (bpm)	100	114
SpO_2_ (%)	96	91
Arterial blood gas analysis		
pH	7.42	7.29
PaO_2_ (mmHg)	207	167
PaCO_2_ (mmHg)	40	57
EB (mEq/L)	1.8	0.4
PaO_2_ (mmHg)/FiO_2_	207	167

ECMO-VA - venoarterial extracorporeal membrane oxygenation; RPM - rotations per minute; FiO_2_ - inspired oxygen fraction; MAP - mean arterial pressure; HR - heart rate; SpO_2_ - blood oxygen saturation; PaO_2_ - arterial oxygen pressure; PaCO_2_ - partial pressure of carbon dioxide; EB - excess base.

The second clinical examination was subsequently performed at the recommended interval. Thus, with a confirmed diagnosis of BD and related legal support, intensive care support was suspended after the family declined to donate the patient’s organs.

## DISCUSSION

This report demonstrates the feasibility of performing an apnea test for the diagnosis of BD in patients on ECMO.

BD, defined as the irreversible cessation of all brain functions, including brainstem reflex activities, is among the neurological complications most feared by teams that use extracorporeal circulatory support devices.^(1)^ Although the situation is challenging given the diagnosis of BD, these support devices become interesting alternatives for maintaining the viability of organs targeted for transplantation.^(3,5,6)^

In addition to the mandatory criteria for the determination of BD,^(4)^ according to Brazilian legislation (CFM Resolution 2.173/2017), we believe that it is necessary to observe additional factors specific to the context of the ECMO apnea test. We describe this process in the section “Practical recommendations”.

The performance of the ECMO apnea test remains a challenge. In the literature, only one Brazilian reference was found describing this experience; it focused on the context of venovenous ECMO (VV-ECMO), a procedure that has its own particularities, such as the possibility that support will be interrupted by clamping the cannulas, and was published before the current revision of the national legislation for the diagnosis of BD.^(7)^

Considering the practical aspects of the apnea test, which is conducted to determine the absence of respiratory drive in the presence of PaCO_2_ elevation beyond the threshold considered necessary to stimulate the respiratory center, a retrospective review stated that the test is “very difficult to perform” due to the absence of “standardized protocols”.^(4^^.^^8)^

In addition to the increase in PaCO_2_, ensuring adequate oxygenation during the test is also a challenge, especially for conditions that require high ventilatory support, such as ARDS, in which the contribution of patient’s lungs to oxygenation is reduced.^(1)^ In these situations, in addition to adequate preoxygenation, it is necessary to optimize the extracorporeal flow.^(1)^ In the present case, while the patient remained on ECMO, changes in ventilatory parameters were necessary for hemodynamic and ventilatory stabilization. The levels of arterial oxygen pressure (PaO2) during the period ranged from 144 to 209mmHg, with no associated neurological repercussions.

Thus, when performing the apnea test, it is recommended to provide oxygen supplementation (FiO2 = 1.0) through the ECMO device by keeping the individual under IMV in continuous positive airway pressure (CPAP) mode to prevent alveolar derecruitment; alternatively, in cases where pressurization of the respiratory system is not essential, oxygen supply (4 - 6L/minute) can be provided through a suction catheter inserted directly into the ventilatory prosthesis or a circuit connected to a T tube.^(1^^.^^8)^

In this case, in accordance with legislation, we chose to provide oxygen supplementation via ECMO, as recommended in the literature, through the introduction of a tracheal suction catheter (6L/minute) via an orotracheal tube.^(4)^ However, the patient presented significant hypoxemia in the first minute of the apnea test, making it necessary to increase the ECMO flow until reasonable oxygenation values for performing the apnea test were reached (flow: 2.2 - 4.0L/minute), with immediate recovery of this parameter. Subsequently, in the case review process, we considered that CPAP could be used as the supplementary modality since resolution 2.173/2017 recommends it in cases of hypoxia. However, the risk of hypoxemia was minimized due to ECMO support when the sweep flow was titrated in proportion to the blood flow.

In addition, during the test, it is imperative to ensure hemodynamic stability, which is potentially disadvantaged by hypercapnic acidemia secondary to the procedure itself.^(1)^ With VV-ECMO, native cardiac function is preserved and, with volemic assistance and vasoactive drugs, can maintain stability.^(1)^ However, in the VA modality, the individual is partially or totally dependent on ECMO, so extracorporeal assistance should be optimized.^(1)^ Contrary to expectations, in the present case, there was no hemodynamic instability, which may be due to the recovery of ventricular function associated with increased extracorporeal flow for the management of hypoxemia.

Finally, attention should be paid to reducing the excessive elimination of carbon dioxide by ECMO. It is known that if the blood flow rate is kept constant, the removal of carbon dioxide is proportional to the flow of the sweeper.^(1^^.^^8)^ Thus, reducing the sweep flow (0.5 - 1.0L/minute) has been suggested to reach the threshold necessary for confirmation of the apnea test.^(1^^.^^9)^ However, if the flow is reduced to zero, oxygenation may be affected; this can be corrected by increasing the sweeper or by adding exogenous carbon dioxide to the ECMO circuit.^(1)^

The Extracorporeal Life Support Organization (ELSO) refers to the study by Giani et al. and recommends that the individual be under CPAP and the sweeper flow be titrated to a maximum of 1L/minute. If PaCO_2_ does not rise above 60mmHg (or 20mmHg above the pretest value), the sweep flow should be progressively reduced to 0.1L/minute while maintaining adequate oxygenation.^(9)^

Based on reported international experience, the sweep in this study was reduced to 0.5L/minute, and the threshold necessary to confirm the absence of respiratory drive, as required by legislation in Brazil (> 55mmHg), was reached after the apnea test had been run for 10 minutes.^(4)^ When the threshold required for confirmation of the test cannot be reached, BD may not be diagnosable, as there is no national reference with legal alternatives such as the exogenous administration of carbon dioxide, which is a real alternative in international settings.

The aforementioned case reinforces that ECMO should be provided in reference centers by teams trained in the indications for its use and management of the device. This case demonstrates that the diagnosis of BD with the apnea test is possible in this context, although the expertise of the team is necessary.

### Practical recommendations

1. Consider the effect of ECMO flow and, eventually, renal replacement therapy (RRT) to estimate the clearance of sedative drugs and the appropriate time to start the diagnostic evaluation of BD.
2. At bedside, consider the proportionality of the physiological contribution of ECMO (hemodynamic, respiratory or mixed) based on the knowledge of applied physiology and the possible use of objective measures (ultrasound, for example) to then define the most appropriate parameter adjustment strategies for each patient.
3. Ten minutes before the collection of the first blood sample for arterial blood gas analysis, in addition to the recommended ventilatory adjustments, additional preoxygenation should provided via ECMO (FiO_2_ = 1.0), and at the beginning of the test, the sweep flow should be reduced to 0.5 - 1.0L/minute.
4. Consider the pulmonary function to select the relevant type of oxygen supplementation (6 L/minute oxygen suction catheter or CPAP, ventilator or positive end-expiratory pressure valve (PEEP)): if pulmonary function is preserved (i.e., ECMO is being used exclusively for hemodynamic support), any of the described modalities can be used. However, in the presence of pulmonary dysfunction (i.e., ECMO is being used for respiratory support, with or without associated hemodynamic support), the CPAP method is recommended.
5. If hypoxemia is observed during the apnea test despite oxygen supplementation, the ECMO flow (rpm) can be increased until adequate blood oxygen saturation (SpO_2_) is achieved, and/or the sweep flow can be increased.
6. If, despite the absence of respiratory movements (apnea), the recommended PaCO_2_ threshold (> 55 mmHg) is not reached, consider repeating the test under exogenous carbon dioxide supplementation of the ECMO circuit. However, because we do not know the national experience, we suggest caution when doing so and, if necessary, consulting teams with considerable expertise on the subject.

## CONCLUSION

This report presents strategies that enable the use of the apnea test for diagnosing brain death in patients undergoing extracorporeal membrane oxygenation. These strategies include arterial preoxygenation via extracorporeal membrane oxygenation combined with titration of the inspired oxygen fraction on the mechanical ventilator; reducing the sweep flow to levels between 0.5 - 1.0L/minute at the beginning of the test and maintaining the blood flow value in the extracorporeal membrane oxygenation, making adjustments only in cases of instability (of hemodynamics and/or oxygenation). Despite the practical challenges, the present case confirms that the execution of the apnea test for the diagnosis of brain death under extracorporeal membrane oxygenation is feasible.
